# Neutrophil Extracellular Trap Formation Is Independent of *De Novo* Gene Expression

**DOI:** 10.1371/journal.pone.0157454

**Published:** 2016-06-16

**Authors:** Gabriel Sollberger, Borko Amulic, Arturo Zychlinsky

**Affiliations:** Department of Cellular Microbiology, Max Planck Institute for Infection Biology, Berlin, Germany; University of Tübingen, GERMANY

## Abstract

Neutrophils are essential innate immune cells whose responses are crucial in the clearance of invading pathogens. Neutrophils can respond to infection by releasing neutrophil extracellular traps (NETs). NETs are formed of chromatin and specific granular proteins and are released after execution of a poorly characterized cell death pathway. Here, we show that NET formation induced by PMA or *Candida albicans* is independent of RNA polymerase II and III-mediated transcription as well as of protein synthesis. Thus, neutrophils contain all the factors required for NET formation when they emerge from the bone marrow as differentiated cells.

## Introduction

Neutrophils (also called polymorphonuclear leukocytes, PMNs) are essential for innate immune defense because they are directly antimicrobial and can shape adaptive immunity [1, 2]. Neutropenic individuals are prone to infections, underscoring the key role of neutrophils in fighting pathogens. PMNs differentiate in the bone marrow and are released in high numbers into the circulation as terminally differentiated cells. During infections neutrophils are rapidly recruited to inflammatory sites where they activate different antimicrobial programs, such as phagocytosis, production of reactive oxygen species (ROS), degranulation or the formation of neutrophil extracellular traps (NETs). NETs are released by PMNs after the activation of a specialized cell death pathway and consist of chromatin bound to cytoplasmic proteins [3, 4, 5]. Microorganisms as well as chemical compounds trigger NET formation, however, the molecular mechanism leading to release of NETs is only poorly characterized. Many NET inducers trigger MAP kinase signaling [6], activate NADPH oxidase (Nox2) and involve the subsequent production of ROS. This leads to granule rupture mediated by a protein complex called “azurosome”, translocation of neutrophil elastase (NE) to the nucleus, chromatin decondensation and NET production [7]. NETs sequester and immobilize pathogenic organisms, thus contributing to immune defense. Furthermore, NETs are dyresgulated in several auto-immune and inflammatory diseases, making them an important target for potential therapeutic interventions [5].

Most proteins required for neutrophil antimicrobial activity are transiently synthesized during development and packed in specialized granules that are deployed upon PMN activation. Indeed, essential neutrophil antimicrobial defense proteins like NE, Proteinase 3, Cathepsin G or Myeloperoxidase (MPO) are only produced during a neutrophil precursor stage and not in circulating cells [8]. Despite this, PMNs respond to bacteria by markedly changing gene expression patterns [9]. Most prominently, they produce chemokines like Interleukin (IL)-8 or Macrophage Inflammatory Protein (Mip) -1α. Furthermore, the MAP kinase pathways, which are known to be essential for NET induction, can also induce transcription. We therefore tested whether transcription or translation are required for NET formation.

## Materials and Methods

### Inhibitors

Actinomycin D (Sigma), flavopiridol (Enzo Life Sciences), CAS 577784-91-9 (Merck Millipore), cycloheximide (Sigma)

### Isolation and stimulation of human neutrophils

Neutrophils were isolated from blood of healthy volunteers and according to the Declaration of Helsinki. Study participants provided written informed consent. All samples were collected with approval from the ethics committee–Charité –Universitätsmedizin Berlin. After centrifugation over Histopaque-1119 (Sigma), neutrophils were purified over a discontinuous Percoll gradient [4]. Experiments were performed in RPMI-1640 (w/o phenol red) supplemented with 10 mM HEPES and 0.2% human serum albumin. Cells were seeded at 10^5^/well (96 well plate) for Mip-1α ELISA or 2x10^5^/well (24 well plate) for NET experiments and incubated with transcription/ translation inhibitors for 5 min before treatment with LPS (1 g/ ml, from *Salmonella typhimurium* (Enzo)), PMA (100 nM) or infection with *Candida albicans* hyphae (opsonized with 10% human serum, 30min at 37°C, before infection).

### Isolation and stimulation of murine neutrophils

Mouse breeding and experiments were approved by the Berlin state authority Landesamt für Gesundheit und Soziales. All animals were locally bred at the Max Planck Institute for Infection Biology. Animals were housed in approved specific pathogen free (SPF) conditions, maintained on a 12 hour light/dark cycle and fed *ad libitum*. Precautions were taken to minimize suffering of the animals. We used male mice that were 8–12 weeks of age. Group size was n = 3. Mice were sacrificed by cervical dislocation the night before the experiment and tibias and femurs were stored at 4°C overnight. Neutrophils were purified from bone marrow by negative selection using EasySep™ Mouse Neutrophil Enrichment Kit (Stemcell Technologies). NETs were induced by plating 10^5^ cells in tissue culture plates (24 wells) in RPMI-1640 supplemented with 10 mM Hepes, 2% mouse serum (Dnase -/-) and 100 ng/ml murine GCSF. Stimulation was with PMA (100 nM) or heat killed *C*. *albicans* at a MOI of 3, both for 15 hours. *C*.*albicans* yeast were grown for 3h at 37°C to induce hyphal growth, followed by 1h incubation at 65°C for heat inactivation. Cytokine production was assayed in RPMI-1640 supplemented with 10% FCS after activation with 200 ng/ml LPS.

### ELISA/ LDH

Human and mouse Mip-1α levels in culture supernatants were determined using BD DuoSet ELISA kit (R&D). LDH release was quantified from the same supernatants by Cytotox 96 Non-Radioactive Cytotoxicity Assay (Promega).

### Immunofluorescence microscopy

Cells were fixed with 2% paraformaldehyde (PFA) and processed as previously reported [10]. NETs were detected with mouse mAB PL2-3, directed against the subnucleosomal complex of Histone 2A, Histone 2B, and chromatin [11] and an anti-NE antibody (Calbiochem). Hoechst or DAPI were used to counterstain DNA. Image acquisition was on a Leica DMR upright fluorescence microscope equipped with a Jenoptic B/W digital microscope camera.

### NET quantification

NETs were enumerated using three different techniques: 1. a SYTO/SYTOX staining technique. The cell impermeable SYTOX orange dye (1 μM) was used to detect NETs. SYTO green, a cell-permeable DNA dye (250 nM), was used to determine the total number of cells. Images were taken on a Leica DM IRBE inverted microscope. 2. Immunofluorescence staining as described above, followed by automated quantification of NETs using ImageJ. The Hoechst signal was used to calculate the total amount of cells per microscopic field and NETs were quantified by the PL2-3 signal (chromatin) accounted for nuclear expansion [10]. 3. By using the cell impermeable DNA dye SYTOX green (50 nM) and measuring in a fluorometer with an excitation/ emission of 485/518 nm, respectively.

### Quantitative real time PCR

RNA was isolated using RNeasy mini kit (Qiagen) and cDNA was synthetized with the High-capacity RNA-to-cDNA kit (Applied Biosystems) according to manufacturer’s protocol. Real-time PCR was performed on StepOnePlus Real-Time PCR System with 2x Fast SYBR Green master mix (Applied Biosystems). Previously verified primers [12] were used for MIP-1α: F 5’-AGCTGACTACTTTGAGACGAGCA-3’ and R 5’-CGGCTTCGCTTGGTTAGGA-3’, and the housekeeping gene β2-microglobulin: F 5’-CTCCGTGGCCTTAGCTGTG-3’ and R 5’-TTTGGAGTACGCTGGATAGCCT-3’. Standard curves were used to establish amplification products. Data were analyzed using StepOne software and expressed as relative amount of MIP1-α product divided by relative amount of β2- microglobulin product for each treatment.

### Statistical Analysis

**Fig 1:**
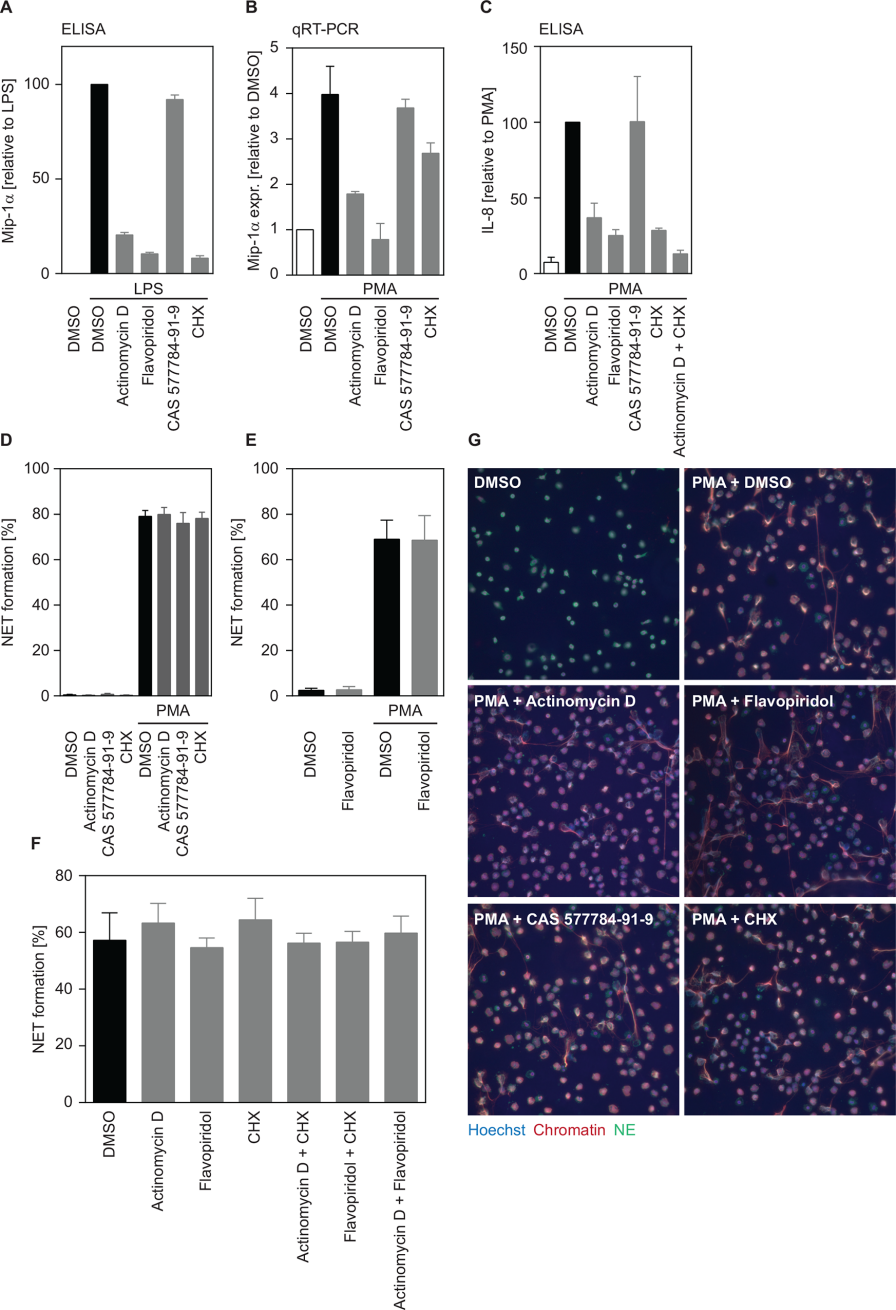
Human neutrophils do not require transcription or translation to release NETs in response to PMA. (A-G). Inhibitors were used at the following concentrations: Actinomycin D (1 μg/ ml), flavopiridol (0.05 μM), CAS 577784-91-9 (10 μM), CHX (1 μg/ ml). (A) Actinomycin D, flavopiridol and CHX, but not CAS 577784-91-9 inhibit *de novo* production of Mip-1α induced by LPS. (B) Actinomycin D and flavopiridol, but not CAS 577784-91-9or CHX block mRNA transcription of PMA-treated human neutrophils. (C.) Actinomycin D, flavopiridol and CHX, but not CAS 577784-91-9 inhibit *de novo* production of IL-8 induced by PMA. (D-G) Actinomycin D, flavopiridol, CAS 577784-91-9 or CHX do not block NET formation in response to PMA (100 nM). (D, E). NET formation was quantified by adding the DNA dye SYTO green (total cells) and the cell impermeable DNA dye SYTOX orange (NETs). (F) NET formation was quantified by immunofluorescence staining of chromatin and Hoechst as previously described [10]. Graphs show mean ± SEM from independent experiments with at least 3 different donors. (G) Representative images of human neutrophils stained with PL2-3 (chromatin, red), NE (Neutrophil Elastase, green) and Hoechst (blue) after treatment with transcription or translation inhibitors and PMA induction. (D-F) Statistical analysis revealed no significant changes after inhibitor treatment.

Statistical significance was determined using one way analysis of variance (ANOVA) with multiple comparison, comparing all inhibitor treatments to DMSO. An exception is Fig 1E, where an unpaired T test was used, comparing DMSO to flavopiridol treatment. A statistically significant result was considered to be p<0.05. Raw values from individual donors and mice for all cytokine and NET quantifications are included as supplementary excel files (S1 Table and S2 Table).

## Results

To investigate the requirement of transcription or translation for NET formation, we treated human primary neutrophils with the RNA polymerase II inhibitors actinomycin D and flavopiridol, the RNA polymerase III inhibitor CAS 577784-91-9 and the translation inhibitor cycloheximide (CHX). We first confirmed the activity of our inhibitors and titrated the minimal concentrations required for inhibition in neutrophils. To accomplish this we used as our readout the transcription and translation of Mip-1α, a chemokine known to be produced *de novo* in response to LPS [13] (Fig 1A, S1A Fig). Both RNA polymerase II inhibitors as well as CHX efficiently blocked *de novo* production of Mip-1α in a dose-dependent manner (S1A Fig). As expected, inhibition of RNA polymerase III did not influence Mip-1α production (Fig 1A, S1A Fig). None of the inhibitors were toxic to PMNs at the concentrations used (Fig 1D and 1E, S1B Fig). We next used the same readout with PMA, the stimulus to be used in NET induction, to validate the inhibitory concentrations. Upon PMA treatment, neutrophils induced transcription of Mip-1α mRNA, which was blocked by addition of both actinomycin D and flavopiridol (Fig 1B). When we analyzed production of Mip-1α at the protein level, we were not able to detect any protein after PMA treatment (data not shown). However, PMA treatment of neutrophils led to *de novo* synthesis of the chemokine IL-8, which was inhibited by actinomycin D, flavopiridol and CHX (Fig 1C). These data demonstrate that all inhibitors were able to efficiently block *de novo* gene expression in PMA-treated neutrophils.

We then investigated whether inhibition of transcription or translation affected NET formation of PMA-treated neutrophils. We quantified NET formation with three different methods and found that inhibition of transcription or translation had no impact on NET formation upon PMA induction (Fig 1D–1G, S2A Fig). Addition of actinomycin D and CHX together showed an additive effect on the inhibition of IL-8 production (Fig 1C). Therefore we also treated neutrophils with combinations of inhibitors before PMA treatment. Again, we found no effect on NET formation (Fig 1F, S2C Fig). These data show that human neutrophils do not require *de novo* gene expression to make NETs in response to PMA.

**Fig 2:**
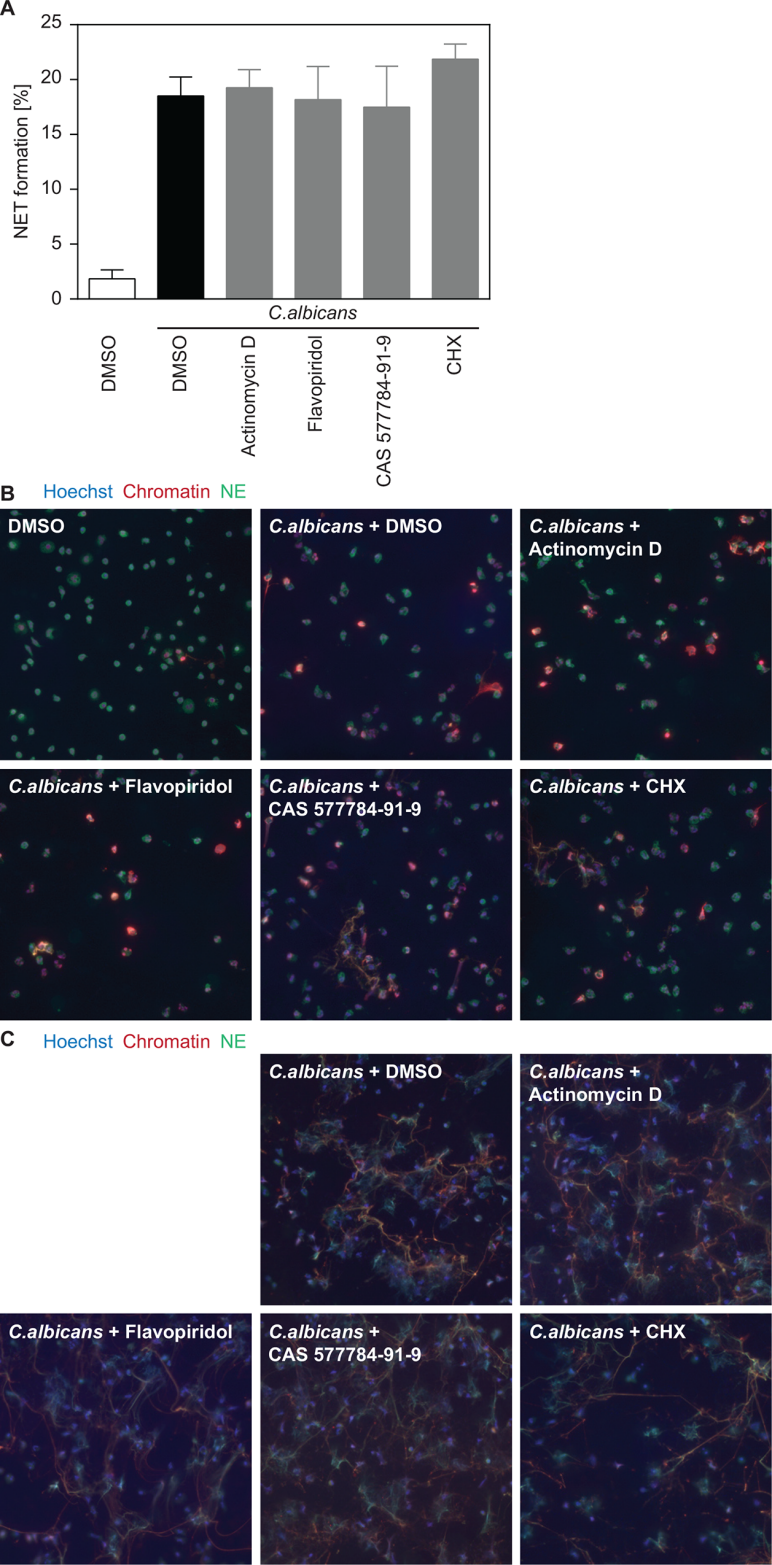
Human neutrophils do not require transcription or translation to release NETs in response to *C*.*albicans*. (A, B) Inhibitors were used at the following concentrations: Actinomycin D (1 μg/ ml), flavopiridol (0.05 μM), CAS 577784-91-9 (10 μM), CHX (1 μg/ ml). Cells were infected with opsonized *C*.*albicans* at MOI 5 and subsequently fixed for immunofluorescence staining. (A) NET formation was quantified at 2h after infection by immunofluorescence staining of chromatin and Hoechst. Graphs show mean ± SEM from independent experiments with 3 different donors. (B) Representative images of an early time point (2h) of human neutrophils infected with *C*.*albicans* and treated with transcription or translation inhibitors. Cells were stained with PL2-3 (chromatin, red), NE (Neutrophil elastase, green) and Hoechst (blue). (C) Representative images of a late time point (4h) of *C*.*albicans*-infected neutrophils showing spread NETs that are unaffected by inhibitors of gene expression. (A) Statistical analysis revealed no significant changes after inhibitor treatment.

We also analyzed the involvement of transcription and translation in NET formation induced by the fungal pathogen *Candida albicans*. Similarly to PMA treatment, infection of human neutrophils with *C*. *albicans* induced the formation of NETs, which occurred independently of transcription and translation (Fig 2A, S2B Fig). Importantly, irrespective of inhibitor treatment, all cells proceeded from early stages of NETosis with decondensed chromatin (Fig 2B) to fully spread NETs (Fig 2C), again demonstrating that transcription or translation inhibitors were not able to block NET formation.

**Fig 3:**
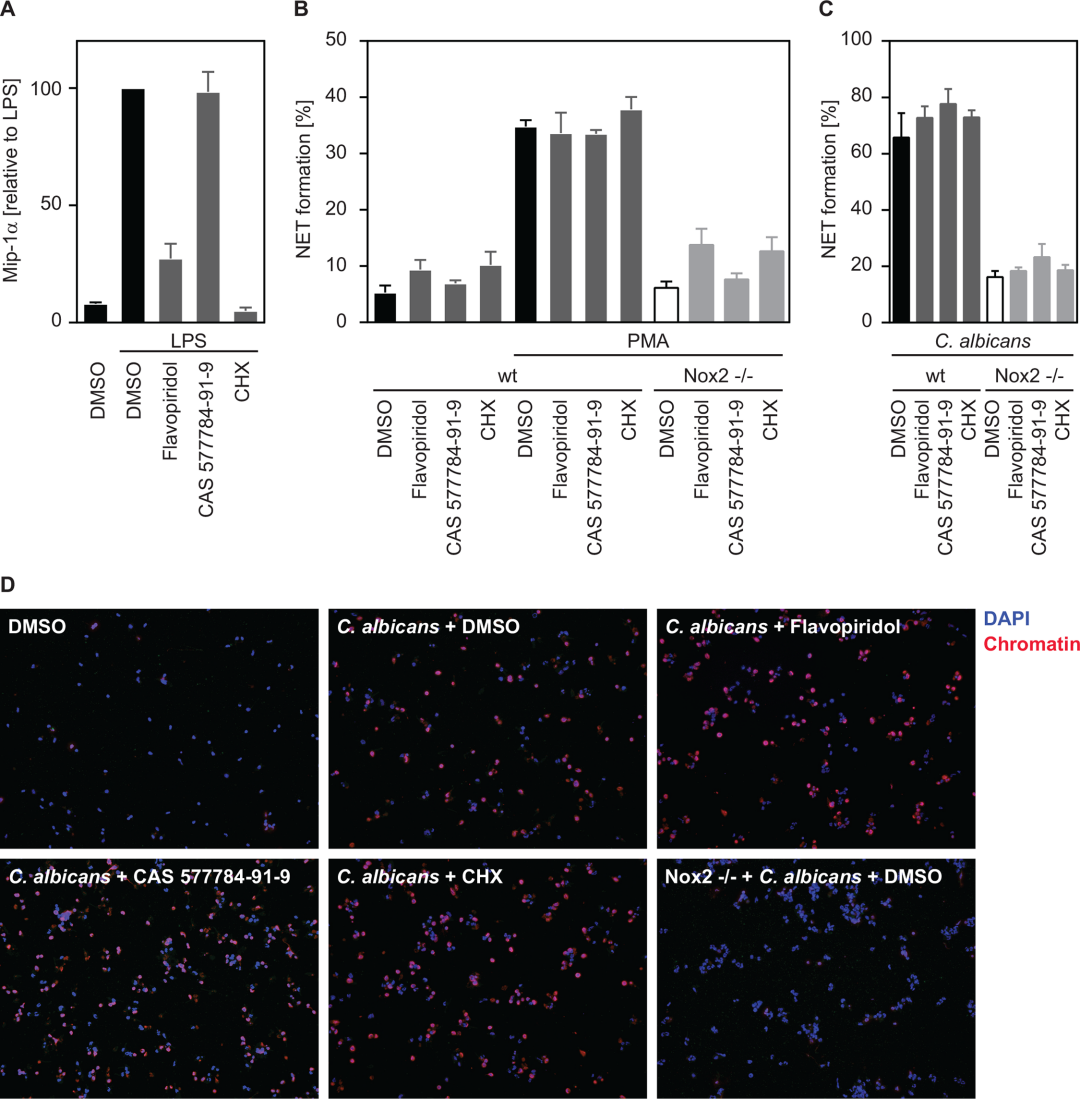
Mouse neutrophils do not require transcription or translation to release NETs. Inhibitors were used at the following concentrations: Flavopiridol (0.05 μM), CAS 577784-91-9 (10 μM), CHX (1 μg/ ml). (A) Flavopiridol and CHX, but not CAS 577784-91-9 block Mip-1α production in response to LPS. (B) PMA-induced NET formation is not inhibited by flavopiridol, CAS 577784-91-9 or CHX. Neutrophils isolated from Nox2 -/-mice are used as negative control. (C) NET formation in response to heat-killed *C*. *albicans* is not blocked by inhibitors. Graphs show mean ± SEM of experiments with neutrophils isolated from 3 mice. (D) Representative immunofluorescence pictures of murine neutrophils stained with PL2-3 (chromatin, red) and DAPI (blue) after infection with *C*. *albicans* (MOI 3) and treatment with transcription/ translation inhibitors. (B, C) Statistical analysis revealed no significant changes after inhibitor treatment.

We then tested an involvement of transcription and translation in murine neutrophils purified from bone marrow. The RNA polymerase II inhibitor flavopiridol as well as CHX blocked production of Mip-1α efficiently (Fig 3A, S1C Fig). Importantly, inhibition of neither RNA polymerase II, RNA polymerase III nor protein synthesis had an impact on NET formation in response to PMA (Fig 3B) or to *C*. *albicans* (Fig 3C and 3D). As previously shown [14], Nox2 -/- neutrophils failed to produce NETs to either stimuli (Fig 3C and 3D) and are here used as a control. Taken together these data demonstrate that NET formation of both murine and human neutrophils occurs independently of *de novo* gene expression and thus relies on proteins already existing in neutrophils.

## Discussion

In line with previously published findings that LPS-induced NETs don't require protein translation for their formation [15], we demonstrate that PMA- and *C*.*albicans*-induced NETs proceed independently of translation. Importantly, we also demonstrate that transcriptional responses are dispensable for NET formation of human and murine neutrophils. Despite the demonstrated efficiency of actinomycin D, flavopiridol and CHX, the possibility remained that minute amounts of *de novo* produced mRNA or protein contribute to NETosis. However, treatment with combinations of inhibitors did not block NET formation either, although they had an additive effect on blocking chemokine production. We therefore conclude that PMA- and *C*.*albicans*-induced NETosis indeed occur independently of *de novo* gene expression.

Notably, human and murine NETs are morphologically distinct; human NETs are spread out, especially after infection with *C*. *albicans*, whereas murine NETs are compact and less diffuse. This makes it challenging to accurately quantify human *C*.*albicans*-induced NETs We thus employed two different quantification approaches. The use of a cell impermeable DNA dye has limitations since the fluorometer readout does not discriminate between different forms of cell death. However, it showed that the overall response to *C*.*albicans* infection was similar in all settings tested. Furthermore, quantification by immunofluorescence staining and analyzing NETs by using an anti-chromatin antibody confirmed that also *C*.*albicans*-induced NETs didn’t require transcription or translation. This finding is important since *C*.*albicans* infection is a more physiological trigger of NET formation than PMA.

NET formation can be induced by various stimuli and, although it might be executed via different pathways, it is also accompanied by induction of transcription. Indeed, MAPK signaling is essential for NET formation and also for the activation of the complex transcriptional program of neutrophils. Importantly, here we described that NETs are formed independently of macromolecular synthesis. Neutrophil activation thus involves parallel pathways: one that relies on transcription to produce chemokines and to amplify the inflammatory response, and a second, transcription-independent one that results in NETs and other antimicrobial effects.

## Supporting Information

S1 Fig Inhibitor titration. (A, B) Increasing doses of inhibitors were added to human neutrophils before LPS stimulation. Actinomycin D: 0.01 μg/ ml; 0.1 μg/ ml; 1 μg/ ml; 5 μg/ ml, flavopiridol: 0.02 μM; 0.05 μM; 1 μM, CAS 577784-91-9: 0.1 μM; 1 μM; 10 μM; 50 μM, CHX: 0.1 μg/ ml; 1 μg/ ml; 10 μg/ ml; 100 μg/ ml. Minimal inhibitor concentrations used for NET experiments are shown in blue. (A) The efficiency of inhibitors was analyzed in human primary neutrophils by measuring their effect on *de novo* production of Mip-1α in response to LPS stimulation (1 μg/ ml) for 20h. (B) The toxicity of inhibitors was monitored by measuring LDH release after 20h of LPS treatment. Graphs show mean ± SEM of independent experiments with 3 different donors. (C) Increasing doses of inhibitors were added to murine neutrophils before stimulation with LPS. Flavopiridol: 0.025 μM; 0.05 μM; 0.1 μM; 0.2 μM; 1μM, CAS 577784-91-9: 1 μM, CHX: 0.5 μg/ ml; 1 μg/ ml; 5 μg/ ml. Minimal concentrations blocking production of Mip-1α and subsequently used for NET experiments are shown in blue. Efficiency of inhibitors was analyzed by measuring production of Mip-1α in response to LPS stimulation (200 ng/ ml) for 20h.
(EPS)

S2 Fig No effect of individual or combined inhibitors on NET formation. Inhibitors were used at the following concentrations: Actinomycin D (1 μg/ ml), flavopiridol (0.05 μM), CAS 577784-91-9 (10 μM), CHX (1 μg/ ml). (A, B) Human primary neutrophils were treated with transcription/ translation inhibitors as indicated. (A) After inhibitor treatment, cells were treated with 50 nM SYTOX green, stimulated with 100 nM PMA and analyzed by measuring emission of SYTOX green every hour in a fluorometer. (B) After inhibitor treatment, neutrophils were treated with 50 nM SYTOX green and infected with opsonized *C*.*albicans* at MOI 5. SYTOX emission was measured every hour in a fluorometer. (C) Representative immunofluorescence pictures of human primary neutrophils treated with combinations of transcription/ translation inhibitors as indicated and stimulated with 100 nM PMA. Cells were stained with PL2-3 (chromatin, red), NE (Neutrophil elastase, green) and Hoechst (blue). (D) Representative pictures of SYTO green/ SYTOX orange assay in human primary neutrophils stimulated with *C*.*albicans* (MOI 5). SYTO green stains all cells, SYTOX orange stains dead cells and extracellular traps.
(EPS)

S1 Table Numerical values used for quantification. (XLSX)

S2 Table Numerical values used for quantification of supplementary data. (XLSX)
